# Relationship between the genetic polymorphisms of *vicR* and *vicK Streptococcus mutans* genes and early childhood caries in two-year-old children

**DOI:** 10.1186/s12903-018-0501-y

**Published:** 2018-03-12

**Authors:** Pei Lin Zhuang, Li Xia Yu, Juan Kun Liao, Yan Zhou, Huan Cai Lin

**Affiliations:** 1 Guangdong Provincial Key Laboratory of Malignant Tumor Epigenetics and Gene Regulation, Department of Stomatology, Sun Yat-Sen Memorial Hospital, Sun Yat-Sen University, 107 Yanjian Road West, Guangzhou, 510120 China; 2 Guangdong Provincial Key Laboratory of Stomatology, Sun Yat-Sen University, 74 2nd Zhongshan Road, Guangzhou, 510055 China; 3 Department of Preventive Dentistry, Guanghua School of Stomatology, Sun Yat-Sen University, 56 Lingyuan Road West, Guangzhou, 510055 China

**Keywords:** Dental caries, Genetic polymorphism, Missense mutation, *Streptococcus mutans*

## Abstract

**Background:**

The VicRK two-component signalling system regulates virulence and cariogenicity in *Streptococcus mutans* (*S. mutans*). The purpose of this study was to explore the genetic polymorphisms of the *vicR* and *vicK* genes, which are associated with dental caries in children with *S. mutans*.

**Methods:**

In this study, 121 (from each group) clinical *S. mutans* strains were isolated from caries-free children and children with high-severity caries to sequence the *vicR* and *vicK* genes. Genomic DNA was extracted from *S. mutans* strains and amplified using PCR. The PCR products were purified and sequenced. A chi-squared test and ABI Variant Reporter software were used to analyse the sequencing results.

**Results:**

The 242 clinically isolated *S. mutans* strains contained the full-length *vicR* and *vicK* genes. No nucleotide sequence insertions or deletions were observed in the two genes. Four silent point mutations were identified in the *vicR* genes, and no missense mutations could be detected. Forty-one mutations were identified in the *vicK* genes. In addition to 32 silent mutations, 9 missense mutations at the 173, 337, 470, 1051, 1132, 1258, 1260, 1277, and 1348 bp positions were found. The distribution frequencies of the missense mutations were not significantly different between the groups, except for the C470T mutation. The frequency of the C470T missense mutation was higher in the high-severity caries group than in the caries-free group.

**Conclusions:**

*vicR* sequences are highly conserved in *S. mutans* clinical isolates. The locus 470 missense mutation of the *vicK* gene may be related to caries in children with *S. mutans.*

## Background

The human oral microbial ecosystem is one of the most complex bacterial communities known. Approximately 700 bacterial species are estimated to exist in the oral cavity [1, 2]. Among these bacteria, *Streptococcus mutans* (*S. mutans*) is one of the few species that have been consistently linked with dental caries [3, 4]. From the initial colonization stages onward, *S. mutans* undergoes continuous dynamic challenges, such as changes in pH, temperature, and nutrient levels, to which it must respond and adapt. Generally, in bacteria, two-component regulatory systems (TCRSs) are used as “molecular switches” in response to environmental changes [5, 6]. These regulatory networks are essential for bacterial adaptation, survival, and virulence.

Based on the genome sequence, 13 TCRSs have been identified in *S. mutans* UA159 [7]. One of these TCRSs, the VicRK signal transduction system, affects various virulence attributes of *S. mutans* [8, 9]*.* The system is composed of a histidine kinase sensor protein (VicK) located in the membrane and a cytoplasmic response regulator protein (VicR). Through phosphorylation reactions, extracellular signals are sensed by VicK, and then the VicK histidine kinase transmits the message to VicR, which modulates gene expression [10, 11].

*vic* genes regulate the expression of several virulence-associated genes that affect synthesis and adhesion to polysaccharides, including *gtfBCD*, *ftf*, and *gbpB* [12]. Moreover, compared with the wild-type UA159 strain, strains without *vicK* form aberrant biofilms, with a reduced rate of total glucan formation [12]. In addition, the inactivation of *vicK* produces a reduced level of lactic acid and enhances the acid tolerance of *S. mutans* [13]; a *vicK* knockout mutant has been shown to be more sensitive to H_2_O_2_ than the wild-type [8].

*A vicR* null mutation is apparently lethal to *S. mutans*. VicR acts directly on the promoter regions of the *gtfB, gtfC*, and *ftf* genes. Overproduction of the VicR transcript upregulates these genes [12]. In addition, VicR binds specifically to the *comC* gene, thereby negatively affecting the transcription of *comC*, *comDE*, *comX*, and *nlmC* [10]. Moreover, genes such as *atlA*, *bmsH*, *glnQ*, *copy*, *wapA*, *relR*, *gcrR*, *plsX*, and *nlmC,* demonstrate direct binding by *S. mutans* VicR. Among these genes, VicR negatively regulates *nlmC* and *copY* and positively regulates *atlA, bmsH* [14].

Early childhood caries (ECC) remains one of the most common chronic diseases of childhood [15, 16], particularly in China. Many reports have stated that *S. mutans* demonstrates a strong relationship with ECC [17, 18]. However, not every individual colonized by *S. mutans* will have dental decay. The DNA loci associated with strains isolated from children with ECC are different from those found in caries-free children [19, 20]. The VicRK system is important to *S. mutans* growth and virulence; therefore, the purpose of this study was to explore the genetic polymorphisms of the *vicR* and *vicK* genes found in *S. mutans*, which are associated with a distinct caries experience in children.

## Methods

### Bacterial strains

This study, which is a continuation of a previous study [21], primarily aimed to explore the connections between the missense mutations of vicR and vicK genes and the severity of caries in children with *S. mutans*. A case-control group study design was used, and the sample was the same as that used in the previous study [21], in which the sample capacities were set after calculation. All statistical tests were two-sided, and the value of α was set at 0.05. The study subjects were selected from our previous study, which was performed in the Huadu District of Guangzhou in South China. The study protocol was approved by the Ethics Committee of Guanghua School of Stomatology, Sun Yat-sen University (ERC-[2012]-13). The procedures used were previously described [21]. Briefly, 121 caries-free children who were *S. mutans-*positive were selected as the caries-free group using a simple random sampling method. Each *S. mutans*-positive sample was numbered in the caries-free group, and then 121 samples were selected according to the random numbers generated by the computer. To determine the between-group genetic differences in the *vicR* and *vicK* genes of *S. mutans*, children who were *S. mutans-*positive and had a distinct caries status (dmft ≥6) formed the high-severity caries group. The dmft score in the high-severity group was based on the category used in a previous study [22]. In this study, one isolated *S. mutans* strain was selected from each child.

### Extraction of chromosomal DNA

Plaque samples from the children were mixed, sonicated and then dispersed to obtain 10^− 3^ dilutions. The *S. mutans* strains were identified according to the colony morphology, and the colonies were tested for their ability to ferment mannitol, sorbitol, raffinose, melibiose, and aesculin as well as for their ability to hydrolyse arginine. The *S. mutans* strains were cultured in 2 ml of brain-heart infusion broth and incubated under anaerobic conditions (85% N_2_, 5% CO_2_, and 10% H_2_) at 37 °C for 18 h. The bacterial cells were harvested by centrifugation at 5000 rpm (~ 2800×g) for 5 min. Cells were resuspended in 5% Chelex100 buffer (containing 1% Tween-20, 0.03% SDS and 1% NP40). To lyse *S. mutans*, the suspension was treated with 10 units of proteinase K (20 mg/ml) at 37 °C for 1 min. The mixture was incubated at 56 °C for 1 h, followed by boiling for 10 min and immediate cooling on ice for 3 min. The DNA sample was collected according to the protocol of TIANamp Bacteria DNA Kit (Tiangen, Beijing, China). The obtained samples were measured with a UV spectrophotometer at 260 nm and 280 nm to detect the quality and quantity of DNA.

### Amplification of the *vicR* and *vicK* genes

*S. mutans* UA159 was used as a reference strain. The total length of the *vicR* gene was 708 bp (Gene ID: 1028759), and the *vicK* gene length was 1353 bp (Gene ID: 1028760). ABI Primer Designer V3.0 was used to design the PCR primers. The *vicR* gene was PCR-amplified using the paired primers 5’-CG*GGATCC*ATGAAGAAAATTCTAATCGTTGACGA-3′ (*BamH*I site italic) and 5’-CCG*CTCGAG*TTAGTCATATGATTTCATGTAATAAC-3′ (*Xho*I site italic). The *vicK* gene was PCR-amplified using the paired primers 5’-CG*GGATCC*ATGACTAATGTGTTTGAATCAAGTC-3′ (*BamH*I site italic) and 5’-CCG*CTCGAG*TCATGATTCGTCTTCATCTTCTTCC-3′ (*Xho*I site italic).

The PCR reaction was performed in a 25 μl reaction volume containing 2.5 μl of 10× PCR buffer, 1.5 mM of MgCl_2_, 0.2 mM of dNTP mix, 0.2 μM of each primer, 100–300 ng of genomic DNA as a template, and 2 U of Platinum® Taq DNA Polymerase (Invitrogen, Carlsbad, CA, USA). The PCR cycling profile included an initial preheating step at 95 °C for 5 min, followed by denaturation at 95 °C for 30 s, annealing at 60 °C for 30 s, and elongation at 72 °C for 50 s. A total of 30 cycles were performed, followed by a final elongation step at 72 °C for 5 min. The PCR amplification product was analysed by electrophoresis in a 1.5% agarose gel.

### Sequencing of the *vicR* and *vicK* genes

The PCR products were purified using a QIAquick Gel Extraction Kit (QIAgen, Hilden, Germany), according to the manufacturer’s protocol. Ultimately, sequencing was performed with a 3730XL DNA analyser platform (Shanghai Life Technologies Biotechnology Company, Life Technologies, Shanghai, China). The PCR product sequences were compared with known *vicR* and *vicK* gene sequences from the UA159 strain in GenBank using Variant Reporter software.

### Statistical analysis

Data analysis was performed using the SPSS 20.0 software. A Chi-square test was used to analyse single nucleotide polymorphisms of the *vicR* and *vicK* genes in *S. mutans* in the high-severity caries group and the caries-free group. A *P*-value < 0.05 for the chi-squared test was considered statistically significant.

## Results

All 242 clinically isolated *S. mutans* strains included the *vicR* and *vicK* genes. No nucleotide sequence insertions or deletions were observed in the two genes.

Amino acid transversion, according to the *vicR* gene codons, is shown in Table 1**.** Four silent point mutations were identified. No missense mutations were found. The distribution frequencies of the silent mutation sites of the *vicR* gene in the high-severity caries and caries-free groups are shown in Table 2**.**

**Table 1:** Transversion of amino acids due to silent mutations, according to the *vicR* gene codons

Base site	UA159		Clinical strains	
	Codon	Amino acid	Codon	Amino acid
112	TTA	Leucine	CTA	Leucine
177	GAC	Aspartic acid	GAT	Aspartic acid
213	AGC	Serine	AGT	Serine
499	TTG	Leucine	CTG	Leucine

**Table 2:** Details of the silent mutation sites of the *vicR* gene in the high-severity caries and caries-free groups

Silent mutation	Caries-free (*n* = 121)		High-severity (n = 121)	
	n (%)		n (%)	
112 T → C†	5	(4.13)	7	(5.79)
177 C → T	6	(4.96)	7	(5.79)
213 C → T	3	(2.48)	3	(2.48)
499 T → C	116	(95.87)	101	(83.47)

†T → C, T represents the 112 locus base in UA159, and C represents the 112 locus base in the clinical strains

Amino acid transversion, according to the *vicK* gene codons, is shown in Table 3**.** A total of 41 mutations were identified. In addition to the 32 silent point mutations, 9 point mutations were found, resulting in missense mutations at the 173, 337, 470, 1051, 1132, 1258, 1260, 1277, and 1348 bp positions. The distribution frequencies of the mutation sites of the *vicK* gene in the high-severity caries and caries-free groups are shown in Table 4**.** The distribution of the C470T missense mutations was higher in the high-severity caries group than in the caries-free group (*P* = 0.002), and the other missense mutation rates in the two groups did not demonstrate any statistically significant differences (Table 5). The C470T missense mutation denotes a C base at the 470th base in the *vicK* gene of *S. mutans* UA159, while a T base is substituted in the *vicK* gene of clinical isolates.

**Table 3:** Transversion of amino acids, according to the *vicK* gene codons

		UA159		Clinical strains	
Mutation	Base site	Codon	Amino acid	Codon	Amino acid
Silent mutations	27	CCC	Proline	CCT	Proline
	105	TAT	Tyrosine	TAC	Tyrosine
	126	AAA	Lysine	AAG	Lysine
	153	TTG	Leucine	TTA	Leucine
	162	GGC	Glycine	GGT	Glycine
	201	GAC	Aspartic acid	GAT	Aspartic acid
	204	TTG	Leucine	TTA	Leucine
	255	ACG	Threonine	ACT	Threonine
	276	GAG	Glutamic acid	GAA	Glutamic acid
	286	CTG	Leucine	TTG	Leucine
	324	TTG	Leucine	TTA	Leucine
	345	AAG	Lysine	AAA	Lysine
	351	ACC	Threonine	ACT	Threonine
	360	AAT	Asparagine	AAC	Asparagine
	474	CCA	Proline	CCG	Proline
	492	CGG	Arginine	CGA	Arginine
	645	GTC	Valine	GTT	Valine
	667	TTA	Leucine	CTA	Leucine
	735	AGC	Serine	AGT	Serine
	829	CTA	Leucine	TTA	Leucine
	834	GAT	Aspartic acid	GAC	Aspartic acid
	918	AAG	Lysine	AAA	Lysine
	924	TAC	Tyrosine	TAT	Tyrosine
	1056	ACG	Threonine	ACA	Threonine
	1062	CAG	Glutamine	CAA	Glutamine
	1074	ACA	Threonine	ACC/ACG	Threonine
	1083	ATC	Isoleucine	ATT	Isoleucine
	1095	TCC	Serine	TCT	Serine
	1215	AAA	Lysine	AAG	Lysine
	1224	GTC	Valine	GTT	Valine
	1269	GAA	Glutamic acid	GAG	Glutamic acid
	1305	AAC	Asparagine	AAT	Asparagine
Missense mutations	173	GAT	Aspartic acid	GGT	Aspartic acid
	337	AGC	Serine	CGC	Arginine
	470	ACG	Threonine	ATG	Methionine
	1051	ATA	Isoleucine	GTA	Valine
	1132	CTT	Leucine	TTT	Phenylalanine
	1258	GAG	Glutamic acid	AAG	Lysine
	1260	GAG	Glutamic acid	GAC	Aspartic acid
	1227	ACC	Threonine	ATC	Isoleucine
	1348	TCA	Serine	CCA	Proline

**Table 4:** Details of the silent mutation sites of the *vicK* gene in the high-severity caries and caries-free groups

Missense	Caries-free (n = 121)		High-severity (n = 121)	
mutation	n (%)		n (%)	
27 C → T †	7	(5.79)	2	(1.65)
105 T → C	2	(1.65)	3	(2.48)
126 A → G	30	(24.79)	44	(36.36)
153 G → A	2	(1.65)	2	(1.65)
162 C → T	6	(4.96)	5	(4.13)
201 C → T	2	(1.65)	1	(0.83)
204 G → A	1	(0.83)	12	(9.92)
255 G → T	2	(1.65)	6	(4.96)
276 G → A	4	(3.31)	3	(2.48)
286 C → T	2	(1.65)	1	(0.83)
324 G → A	2	(1.65)	1	(0.83)
345 G → A	3	(2.48)	1	(0.83)
351 C → T	2	(1.65)	0	(0.00)
360 T → C	85	(70.25)	67	(55.37)
474 A → G	75	(61.98)	70	(57.85)
492 G → A	3	(2.48)	1	(0.83)
645 C → T	2	(1.65)	1	(0.83)
667 T → C	2	(1.65)	1	(0.83)
735 C → T	114	(94.21)	105	(86.78)
829 C → T	100	(82.64)	99	(81.82)
834 T → C	4	(3.31)	5	(4.13)
918 G → A	1	(0.83)	2	(1.65)
924 C → T	3	(2.48)	5	(4.13)
1056 G → A	5	(4.13)	6	(4.96)
1062 G → A	36	(29.75)	16	(13.22)
1074 A → C	33	(27.27)	15	(12.40)
A → G	3	(2.48)	1	(0.83)
1083 C → T	1	(0.83)	2	(1.65)
1095 C → T	4	(3.31)	9	(7.44)
1215 A → G	3	(2.48)	2	(1.65)
1224 C → T	2	(1.65)	1	(0.83)
1269 A → G	7	(5.79)	3	(2.48)
1305 C → T	7	(5.79)	6	(4.96)
1348 T → C	5	(4.13)	3	(2.48)

†C → T, C represents the 112 locus base in UA159, and T represents the 112 locus base in the clinical strains

**Table 5:** The distribution frequencies of the missense mutation sites of the *vicK* gene in the high-severity caries and caries-free groups

Missense	Caries-free (n = 121)		High-severity (n = 121)		*x* ^2^	*P*-value^a^
mutation	n (%)		n (%)			
173 A → G†	0	0.00	3	2.48	3.308	0.247^b^
337 A → C	118	97.52	115	95.04	1.309	0.499^b^
470 C → T	1	0.83	12	9.92	9.836	0.002^a^
1051 A → G	115	95.04	115	95.04	0	1.000^b^
1132 C → T	2	1.65	0	0.00	2.017	0.498^b^
1258 G → A	5	4.13	0	0.00	5.105	0.060^b^
1260 G → C	3	2.48	0	0.00	3.308	0.247^b^
1277 C → T	6	4.96	1	0.83	3.678	0.120^b^
1348 T → C	6	4.96	3	2.48	1.309	0.499^b^

†A → G, A represents the 23 locus base in UA159, and G represents the 23 locus base in the clinical strains

^a^Chi-square test

^b^Fisher’s exact test

## Discussion

The profound effects of genetic polymorphisms of virulence factors on *S. mutans* have been a focus of caries research for many years [23, 24]. The vicRK signal transduction system is essential for *S. mutans* by modulating gene expression. However, all prior studies have focused on constructing mutants of laboratory reference strains, and little is known about the gene variation in clinical isolates and its relationship with caries. Compared to laboratory reference strains, clinical isolates are closer to the true disease state.

Hence, in this study, we sequenced the *vicR* and *vicK* genes of *S. mutans* strains isolated from children with a distinct caries status to analyse the effects of *vicR* and *vicK* polymorphisms on the risk of ECC. Mutational analysis showed that VicR in *S. mutans* plays an essential role in the viability of this bacterium [12]. In our study, all 242 clinical isolates had a full-length *vicR* gene, and no missense mutations were found, which indicated that the *vicR* gene was highly conserved among the clinical isolates. This ubiquitous distribution and sequence conservation of *vicR* among the *S. mutans* clinical isolates may suggest that it plays an important role in *S. mutans* biology.

The overall structure of VicK is a long-rod dimer that anchors four connected domains: HAMP (aa 36–86), PAS (aa 87–198), DHp (aa 199–269) and CA (aa 278–450) [25]. Compared to *S. mutans* UA159, all 242 clinical isolates had a full-length *vicK* gene, and nine missense mutation sites were found. All missense mutation sites identified in the present study were distributed in the HAMP, PAS and CA domains. No missense mutation sites were found in the DHp domain. The statistical analysis of the missense mutations was worthy of attention because these changes could affect protein activity. The statistical analysis showed that the differences in missense mutation rates between the caries-free and high-severity caries groups were not significant, except for that of the C470T mutations. The frequency of the C470T missense mutation was higher in the high-severity caries group than in the caries-free group. The cytosine (C) to thymine (T) mutation at the 470th bp in the *vicK* gene leads to a mutation of threonine (T) to methionine (M) at the amino acid residue at position 157 in the VicK amino acid sequence. The 157-amino acid residue belongs to the PAS domain. The PAS domain is a major sensor, with adopted canonical folds and dyad symmetry. The effect of the mutation at site 470 (aa 157) on the activity and function of the VicK protein requires further study.

The present study has some limitations. First, a dmft level of ≥6 was set as the cut-off value, which may have narrowed the selection of candidates for the high-severity caries group. However, this experiment was exploratory in nature. Children with a distinct caries status were utilised to explore and compare the genetic polymorphisms in the *vicR* and *vicK* genes of *S. mutans*. Second, the sample size was relatively small; therefore, a larger sample size may be required for additional between-group analysis.

## Conclusions

The present study provided knowledge of the genetic diversity of the *vicR and vicK* genes of *S. mutans* in children with two distinct caries experiences: caries-free and high-severity caries. The results indicated that in children, sequences of *vicR* are highly conserved in *S. mutans* clinical isolates and the C470T missense mutation of the *vicK* gene may be related to caries experience with *S. mutans.*

## Abbreviations

dmft: Decayed, missing and filled teeth
DNA: Deoxyribonucleic acid
ECC: Early childhood caries
PCR: Polymerase chain reaction
S. mutans: Streptococcus mutans
TCRSs: Two-component regulatory systems
